# Toxicity Evaluation of Individual and Mixtures of Nanoparticles Based on Algal Chlorophyll Content and Cell Count

**DOI:** 10.3390/ma11010121

**Published:** 2018-01-12

**Authors:** Kyung-Seok Ko, Dong-Chan Koh, In Chul Kong

**Affiliations:** 1 Groundwater Department, Geologic Environment Division, Korea Institute of Geoscience and Mineral Resources (KIGAM), Daejeon 34132, Korea; kyungsok@kigam.re.kr (K.-S.K.); chankoh@kigam.re.kr (D.-C.K.); 2 Department of Environmental Engineering, Yeungman University, Kyungbuk 38541, Korea

**Keywords:** algal growth, chlorophyll content, cell count, nanoparticles, toxicity

## Abstract

The toxic effects of individual and binary mixtures of five metal oxide nanoparticles (NPs) were evaluated based on changes in two endpoints of algal growth: the cell count and chlorophyll content. Various effects were observed according to the concentration tested and type of NPs, and there were no significant differences in findings for the two endpoints. In general, ZnO NPs caused the greatest inhibition of algal growth, and Fe_2_O_3_ NPs the least. The EC_50_ for ZnO was 2.0 mg/L for the cell count and 2.6 mg/L for the chlorophyll content, and it was 76 and 90 mg/L, respectively, for Fe_2_O_3_. The EC_50_ values were in the order ZnO > NiO > CuO > TiO_2_ > Fe_2_O_3_. Subsequently, the effects of 30 binary mixture combinations on the chlorophyll content were evaluated. Comparisons were made between the observed and the expected toxicities calculated based on the individual NP toxicities. Overall, additive action (67%) was mainly observed, followed by antagonistic (16.5%) and synergistic (16.5%) actions. These results suggest that environmental exposure to NP mixtures may cause toxicity levels similar to the sum of those of the constituent NPs.

## 1. Introduction

Increasing numbers of commercial nanoparticle (NP) products are being applied in many fields, such as electronics, textiles, medical devices, cosmetics, wastewater technology, and environmental remediation [1]. For example, ZnO NPs are used in dentistry as an antibacterial and in sunscreens, CuO NPs are used in antimicrobial textiles and antifouling paints, and NiO NPs are used in nano devices [2,3]. In general, NPs are classified into four groups: composites, dendrimers, carbon-based, and metal-based materials (e.g., metal oxides) [4,5]. Accidental or intentional release into the environment has occurred with the increased production and use of NPs, and potential ecological effects of metal-based NPs have attracted considerable attention [6,7]. The distinct physical and chemical properties of NPs, such as nano-size, surface characteristics, reactivity, conductivity, and optical properties, are often related with negative ecological toxic effects in environment and unexpected health hazards [8,9].

With the increasing release of NPs into the environment, it is essential to assess the toxicity of NPs using various test organisms [2,10]. To evaluate their environmental impact accurately, it is necessary to adopt appropriate organisms, endpoints, and methods for these assessments, as well as to understand the effects of NP mixtures. Various types of organisms, such as bacteria, algae, protozoa, plants, and fish, have been used to evaluate the toxic effects of pollutants [11]. Research has demonstrated the toxicity of NPs using bioassays [12,13]. For example, the widely used NPs TiO_2_ and ZnO have toxic effects on the growth of microalgae *Pseudokirchneriella subcapitata* (EC_50_; 5.83 mg/L TiO_2_ and 0.042 mg/L ZnO), *Daphnia magna* (EC_50_; ~2000 mg/L TiO_2_ and 3.2 mg/L ZnO), *Vibrio fischeri* (EC_50_; >2000 mg/L TiO_2_ and 1.9 mg/L ZnO), and *Thamnocephalus platyurus* (EC_50_; >2000 mg/L TiO_2_ and 0.18 mg/L ZnO) [14,15]. There are several investigations on the toxicity of silver and platinum NPs and carbon nanotubes on terrestrial animals and bacteria [16,17]. From an eco-toxicological perspective, TiO_2_ and Ag NPs are the most extensively evaluated NPs. Various studies have found a range of toxicity mechanisms (e.g., dissolved ions, interactions with algae, entrapment of algal cells in NP aggregates) for a variety of NPs [18]. Algal growth is one widely used method for evaluating the toxicity of various chemicals because of its high sensitivity, simplicity, and low cost, especially for contaminants introduced into aqueous environments. Algae, which are important aquatic organisms in environments, have been adopted as representative organisms to examine the toxicity of NPs [19,20].

In general, many toxic studies have performed on single (individual) chemicals under controlled (laboratory) conditions rather than under conditions with complex mixtures of chemicals [21]. In reality, the toxic effects of mixtures reflect environmental pollution more realistically. It is, thus, important to understand if the behavior and toxicity of mixture materials can be predicted based on their individual behavior and toxicities, or if a different toxicity pattern will be observed due to potential different interactions with biotic compartments [22]. However, evaluating the response to more than one chemical is a considerably difficult task in the assessment of a contaminated environment [23]. Dissolution and aggregation-agglomeration are the two main processes that can strongly influence the state of metal-based NPs present in suspensions and consequently impact the bioavailability, uptake, and toxicity of NPs [24]. Solubilized metal ions or insoluble inorganic particles produced by metal-based NPs inevitably drive the partial toxicity of NPs to organisms [24]. To improve on this, two basic types of mixture model are general adopted, namely, theoretical models of response (effects) addition and concentration addition. The expected toxic effects of binary mixtures can be determined simply using a mathematical model of probability theory [25] or by EC_50s_ using the toxic unit (TU) base [26,27,28]. The mixture toxic effect can be defined as additive, synergistic (>additive), or antagonistic (<additive) actions based on the results of these models.

The main objective of this investigation was to examine the toxic effects of binary mixtures of five NPs (ZnO, CuO, NiO, TiO_2_, and Fe_2_O_3_) by measuring the chlorophyll content during algal growth. Experiments for the binary mixtures were based on the toxic effects of individual NPs in two endpoints (cell count and chlorophyll content) of algal growth.

## 2. Materials and Methods

### 2.1. Toxic Effects of Individual NPs on the Activity of Algal Growth

The toxicity of the NPs was assessed using the green algae species *Chlorella vulgaris* (KCTC AG10002), which was obtained from the Korean Culture and Tissue Collection. The algae were cultured for three days in BG11 medium at 30 °C and 150 rpm under 5000 lux, and then diluted to a final OD_730_ of 0.3 for test. For the NP inhibition test, 1 mL of the NP solution was exposed to 19 mL of the algal culture (three days at 30 °C and 150 rpm). For the binary mixture tests, 30 combinations (three concentrations of each NP) were examined based on the results for single NPs. Each combination was examined in triplicate (Table 1). Growth inhibition, assessed by measuring the cell count and chlorophyll content, was determined for the single tests after a 72-h incubation period; only the chlorophyll content was measured for the binary mixture tests. Chlorophyll was extracted with 90% (*v*/*v*) acetone and determined using modified standard methods [29]. Following equation was used for the calculation of the chlorophyll content using UV/VIS spectrophotometer (Shimadzu 1240 UV mini, Seoul, Korea).

Chlorophyll (mg/m^3^) = Y × supernatant (mL)/total working volume (L);

Y = 11.64(OD_663_ − OD_750_) − 2.16(OD_645_ − OD_750_) + 0.010(OD_630_ − OD_750_).

The algal cells were counted using a counting chamber (Marienfeld, Lauda-Königshofen, Germany) under an optical microscope.

The EC_50_ (effective concentration of a chemical at which 50% of its effect is observed) values of the NPs were estimated using the program SPEARMAN, which is distributed by the US Environmental Protection Agency. Five NPs were used in this study (size, density, surface area): NiO (30 nm, 6.67 g/cm^3^, 50–100 m^2^/g), CuO (30–50 nm, 6.40 g/cm^3^, 13.1 m^2^/g), TiO_2_ (<25 nm, 3.95 g/cm^3^, 75–85 m^2^/g), and Fe_2_O_3_ (20–40 nm, 5.24 g/cm^3^, 30–60 m^2^/g) from Nanostructured and Amorphous Materials (Houston, TX, USA), and metal oxide ZnO (40–100 nm, 5.61 g/cm^3^, 10–25 m^2^/g) from Alfa Aesar (Tewksbury, MA, USA). The NPs were suspended directly in deionized water (pH 7.8) and dispersed by ultrasonic vibration for 10 min prior to use.

### 2.2. Evaluating the Toxicity of Mixtures of NPs Using Algal Growth

In the toxicity test of binary mixture, the observed toxicity, *P*(*O*), measured in the experiment was compared with the theoretically expected toxicity, *P*(*E*), calculated using a simple mathematical model based on probability theory as *P*(*E*) = *P_x_* + *P_y_* − (*P_x_P_y_*/100): *P_x_* (the inhibition occurred by chemical “*x*”) and *P_y_* (the inhibition occurred by chemical “*y*”) [25,30]. The toxic result was determined synergistic or antagonistic effect if *P*(*O*) was considerably higher or lower (*p* < 0.05 level; null hypothesis), respectively, than *P*(*E*). In contrast, the interaction of binary mixture was considered as additive effect only if the difference between *P*(*O*) and *P*(*E*) was not significant (*p* > 0.05 level). The 95% level of significance was calculated using the Student’s *t*-test (http://www.graphpad.com).

## 3. Results and Discussion

### 3.1. Toxic Effect of Individual NPs on the Activity of Algal Growth

**Figure 1:**
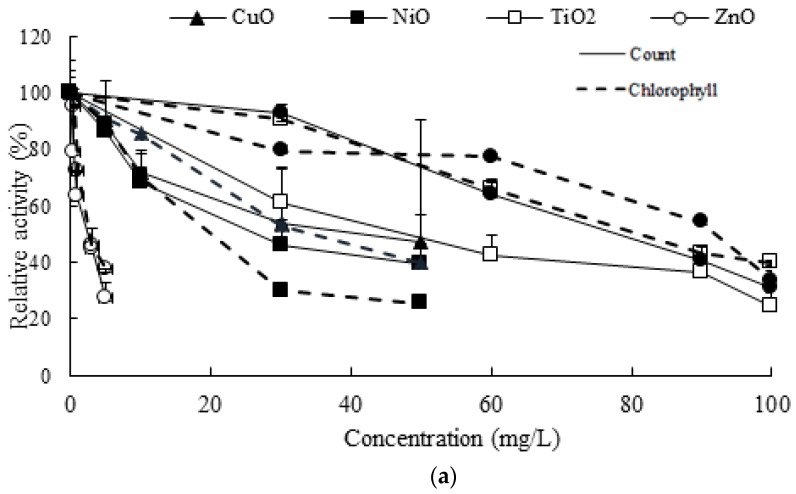

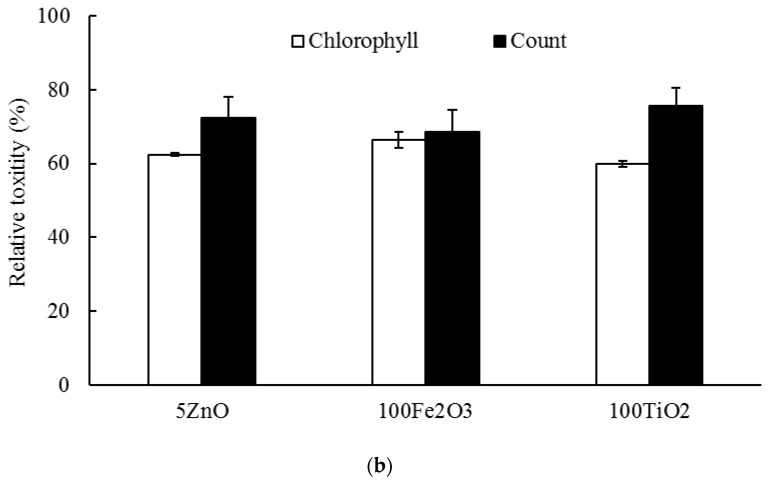
Toxic effects of NPs in two endpoints (cell count and chlorophyll content) of algal growth (**a**). Representative results of the most (5 mg/L ZnO) and least (100 mg/L of Fe_2_O_3_ and TiO_2_) toxic individual NPs (**b**). Comparisons were made based on the relative activity of the control set, 100% activity at no NPs amended condition.

After the preliminary studies, various concentration ranges of individual NP were chosen to examine the toxic effects of NPs on the activity of algal growth. The toxic effects on algal growth were examined by measuring the cell count and chlorophyll content. The algal cell count and chlorophyll content without NP exposure were in the range 2.1–3.7 × 10^7^/mL and 24.4–36.6 g/m^3^, respectively, depending on the batch set. Figure 1 shows the relative cell counts and chlorophyll content (%) under the wide concentration ranges of NPs. No growth stimulation was observed with the NP concentrations tested. The most significant toxic effect was observed in the presence of ZnO NPs, showing 38% chlorophyll content (toxicity 62%) and 28% cell count (toxicity 72%) of the control levels at 5 mg/L. For the other NPs, very low or no observable inhibition was appeared at the lowest concentration tested. For example, there was no significant inhibition with 5 mg/L of CuO and NiO, showing over 85% activity of the control (Figure 1). Figure 1b shows the toxic conditions of the most (ZnO) and least (Fe_2_O_3_ and TiO_2_) toxic NPs among tested conditions. Among tested concentration ranges, the highest toxicity levels on the activity of algal growth were approximately in the range of 60–70% of control at 5 mg/L ZnO and 100 mg/L Fe_2_O_3_ or TiO_2_.

**Figure 2:**
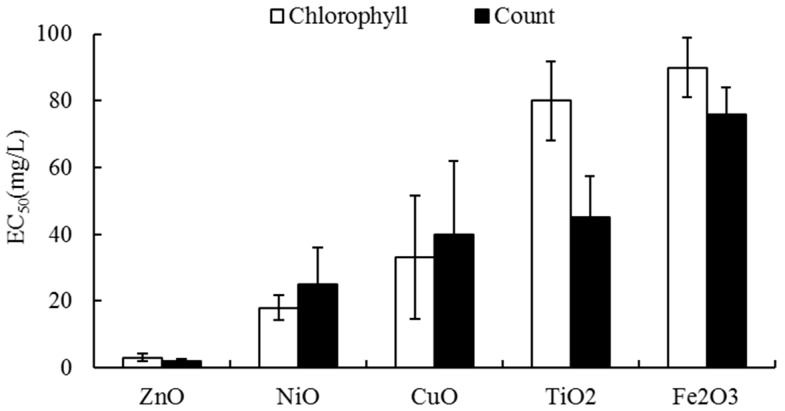
Comparisons of the EC_50s_ of single NPs in two endpoints of algal growth: the cell count and chlorophyll content. The error bar indicates the 95% confidence level.

The toxic effects of the NPs on the activity of algal growth were compared using their EC_50_ values (Figure 2). In the tests with individual NPs, the toxic effects of the NPs on algal growth varied according to the type of NP, with the toxicity ranked in the order ZnO > NiO > CuO > TiO_2_ > Fe_2_O_3_ for both endpoint measurements. The EC_50_ values ranged from 2 (ZnO) to 76 (Fe_2_O_3_) mg/L based on the endpoint of cell count and from 3 (ZnO) to 90 (Fe_2_O_3_) mg/L based on the endpoint of chlorophyll content. Clearly, ZnO caused the greatest inhibition of algal growth. In terms of the EC_50_, the inhibition with ZnO and NiO was approximately 30–38 and 3–5 times that of Fe_2_O_3_ (the weakest inhibition). Slightly lower levels of inhibition (higher EC_50_ values) were observed with the chlorophyll content compared with those determined using the cell count, although the differences in EC_50_ between the two endpoints (cell count and chlorophyll content) were not significant (*p*-values of 0.5537–0.8513). Previously, we found that the sensitivities and toxicity rankings varied depending on the organisms tested [10]. For example, the EC_50s_ for bacterial bioluminescence were ZnO 1.05, CuO 54, NiO 198, Fe_2_O_3_ > 1000, and TiO_2_ > 1000 mg/L, whereas those for lettuce seed germination were ZnO 10.8, CuO 0.46, NiO 17.2, Fe_2_O_3_ > 1000, and TiO_2_ > 1000 mg/L. Previous results also reported that the toxic effects of NPs on the activity of seed germination differed with the seeds tested. Therefore, to assess NPs fully, future studies should examine toxicity using more than one method. Lin and Xing [31] examined the inhibition of ZnO NPs on the activity of seed germination, with EC_50_ values in the range of 20–50 mg/L, which was much less sensitive than in this study of *Chlorella vulgaris*. ZnO NPs were lethal to *Daphnia* in the range 0.89–1.02 mg/L [19], depending on the particle size, which was more sensitive than the effects on algal growth in the current study. Many reports have explained that the toxic effects of metal oxide NPs are mainly caused by the solubilized metal ions [15,19,31]. Heinlaan et al. [15], however, suggested that the toxic effects are mainly caused by the intimate contact between the cell and particle. NP type, solubility, morphology (shape), particle size, crystallinity, surface chemistry, residual chemical impurities, and environmental factors (pH, ionic strength, etc.) have all been reported to affect the assessment of NPs [16,32,33]. Ko and Kong [34] reported that for proper assessment of the toxicity of partially soluble NPs, the conventional method, designed for soluble chemicals, needs to be performed under modified conditions because of their insolubility. In addition to these factors affecting toxicity, other adopted endpoints (cell count, chlorophyll content, absorbance, ATP content, etc.) for algal growth might be considered when assessing NP toxicity.

### 3.2. Toxic Effects of the Mixtures on Algal Growth

**Figure 3:**
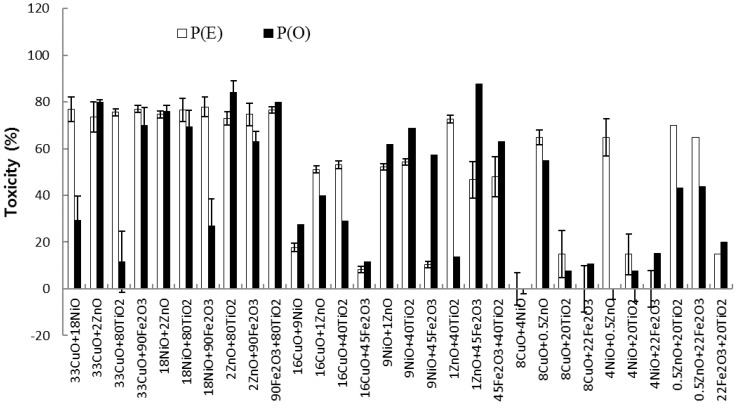
Summary of the observed and expected toxic effects of binary mixtures of NPs based on the chlorophyll contents of algal growth after a 72-h exposure. The term “18NiO + 2ZnO” indicates the final concentrations in the binary mixture, i.e., 18 mg/L NiO and 2 mg/L ZnO.

Toxicity studies examining single chemicals might not provide a complete assessment because the ecosystem is generally contaminated to various mixtures of chemicals. Based on the evaluations of individual NPs, the toxic effects of mixtures of five NPs were observed using equal mixtures of three concentrations of each NP type: 0.5, 1, and 2 mg/L ZnO; 4, 9, and 18 mg/L NiO; 8, 16, and 33 mg/L CuO; 20, 40, and 80 mg/L TiO_2_; and 22, 45, and 90 mg/L Fe_2_O_3_. The three concentrations of each NP (high, intermediate, and low) were mixed pairwise with the respective high, intermediate, and low concentrations of another NP. Chlorophyll content, which was slightly more sensitive than cell count in the presence of CuO and NiO NPs, was used to evaluate the mixture effects for 30 combinations. The control (no added NP) produced a mean chlorophyll level of approximately 26,400 mg/m^3^, whereas algae exposed to binary NP mixtures had chlorophyll levels of 3240–30,200 mg/m^3^, corresponding to 12–114% of the control. Overall, various toxic effects were observed depending on the type of mixture and tested concentrations of the NPs. Figure 3 shows the relative chlorophyll contents of algal cultures exposed to binary NP mixtures. Among the tested conditions, the highest toxicities were observed with the following mixtures, showing a range of 80% to 87% toxicity: 2 mg/L ZnO + 80 mg/L TiO_2_; 1 mg/L ZnO + 45 mg/L Fe_2_O_3_; 2 mg/L ZnO + 33 mg/L CuO; and 90 mg/L Fe_2_O_3_ + 80 mg/L TiO_2_. Most of the mixtures containing ZnO, which was the most toxic NP tested, were very toxic to algal growth. Interestingly, the mixture containing high levels of the least toxic NPs (90 mg/L Fe_2_O_3_ and 80 mg/L TiO_2_) also showed considerable toxicity (80% ± 5.6%). The average toxicities of the mixtures of high, intermediate, and low concentrations of each NP were 59% ± 26.2%, 46% ± 25.4%, and 20% ± 19.8%, respectively. The high concentration mixtures were approximately 1.3 and 3.0 times as toxic as the intermediate and low concentration mixtures, respectively. The same mixtures of NPs, but in different concentrations did not show same trends. Only five out of ten mixtures of NPs (CuO/NiO, CuO/Fe_2_O_3_, NiO/ZnO, NiO/TiO_2_, Fe_2_O_3_/TiO_2_) were generally decreased with decreasing concentration, but others not.

**Figure 4:**
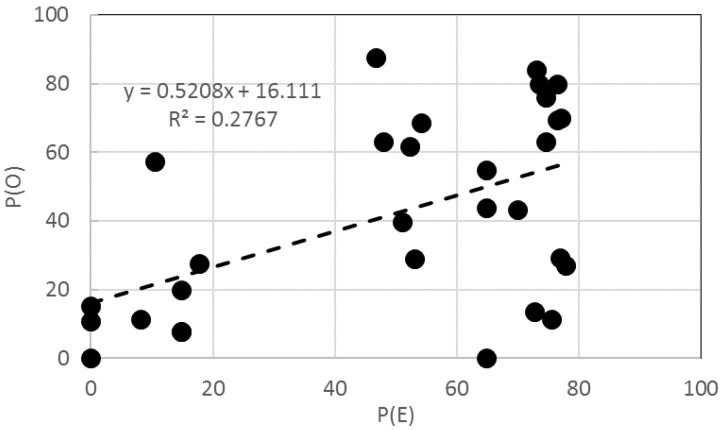
Relationships between the observed and expected toxic effects of 30 binary mixtures of NPs based on the chlorophyll content of algal growth.

The *P*(*O*) of a binary mixture was compared with its *P*(*E*), calculated using the result of individual toxic effects (Figure 3). The ranges of chlorophyll content inhibition for *P*(*O*) and *P*(*E*) were 0–88% (avg. 40% ± 28.3%) and 0–78% (50% ± 29.0%) of the control, respectively. Of the combinations tested, 20 out of the 30 combinations showed additive (observed toxicity similar to expected toxicity) actions, five had synergistic effects, and five had antagonistic effects. No significant correlation (R^2^ = 0.2767) was observed between the results of *P*(*E*) and *P*(*O*) (Figure 4). Although the reasons for the absence of a significant correlation between the results of *P*(*E*) and *P*(*O*) are unclear, the partial solubility of the NPs is one possible explanation. Previous studies observed different modes of toxic action depending on the organisms tested. Synergistic effects (67%) were mainly observed in bacterial bioluminescence tests, whereas both additive (50%) and synergistic (47%) effects were in the activity of seed germination [35]. These results suggested that further studies should examine toxicity using more than one method to assess NPs fully, considering several factors, such as the physicochemical characteristics of the pollutants.

The accurate mechanisms of toxic effects are not largely known, but different mechanisms could cause the toxicity of NPs depending on the organisms tested [36]. Different effects of NP mixtures on plant and animal cells, which may be caused by different features of bioavailability and toxicity across the species, were reported by investigators [22,37]. In general, the toxicity of NPs can be attributed to their chemical properties (e.g., dissolution of ions) or to the oxidative stress or stimuli induced by the physicochemical characteristics of NPs, which result in damage to cellular components [32,38,39]. Lin and Xing [31] reported that the toxic effect of ZnO NPs could be caused by both the solubilized ions and the mere presence of NPs. Several factors could affect the dissolution of metal ions from NPs, such as the concentration, incubation conditions, and type and size of NPs. The toxic mechanism and sensitivity could also vary with the organisms tested. For example, the released Cu ions would have little impact on plants because their toxicity is due specifically to the accumulation of NPs within plant cells [2]. Other studies also found that dissolved metal ions do not account for the total observed toxicity [40]. Therefore, an understanding of the interactions of NPs with the test organisms is important. Research has shown that the toxic effect of NPs is influenced to their morphology, particle size, and bioassays used [12,41]. Brown et al. [42] also reported that small particles are more reactive because of their high specific surface area and ability to penetrate organisms. Axevedo et al. [43] assessed the toxicity of a NP composed by a ZnO and Ag using immobilization and reproduction tests of the model-organism *Daphnia magna*. This mixture study showed an increase in toxicity when compared to the expected (immobilization) and dependent on the concentration used (reproduction), indicating that the toxicity of the mixture can be predicted based on not only the toxicity of their components, but also their interaction between NPs [43]. Since NPs are typically engineered or post-processed for specific applications, their physico-chemical properties and reactivity can vary considerably [44]. However, two key physico-chemical properties of ZnO NPs were suggested that are relevant to or predictive of their high ecotoxicity—The solubility and photoreactivity [44,45].

## 4. Conclusions

In summary, the toxic effects of binary mixtures (NPs) on the activity of algal growth were evaluated after examining the effects of individual exposure. The results of the single exposures were used when designing the mixture exposures. This work indicated the different toxic effects of individual NPs on the two endpoints of algal growth according to the type of NP. Both endpoints (chlorophyll content and cell count) gave similar results. For the binary mixture exposures, additive actions (67%), were predominantly observed, followed by antagonistic (16.5%) and synergistic (16.5%) actions. The toxicity of NPs can show a wide range of variability in laboratory tests. Therefore, the effects of NPs using various methods should be examined under different conditions in further studies. More practically, NPs in the environment exist as mixtures, and they react with the soil and other constituents, which modify their mobility, bioavailability, eco-toxicity, and so on. Although our results suggest that the toxic effects of a mixture are similar to that of the sum of its constituent NPs, more work is needed to clarify the real-time and long-term effects of NPs in environment.

## Figures and Tables

**Table 1:** The three levels of each nanoparticle used in 30 combinations for the study of binary mixture.

NPs	CuO	ZnO	NiO	TiO_2_	Fe_2_O_3_	Combinations
Concentrations (mg/L)	8, 16, 33	0.5, 1, 2	4, 9, 18	20, 40, 80	22, 45, 90	30
